# Prevalence of ADRD Among Medicare Advantage Enrollees in Puerto Rico Compared to US Counterparts

**DOI:** 10.1093/geroni/igaf122.1500

**Published:** 2025-12-31

**Authors:** Maricruz Rivera-Hernandez, Yanru Liao, Yoojin Lee, Michael Crowe

**Affiliations:** Brown University School of Public Health, Providence, Rhode Island, United States; Brown University Center for Gerontology and Healthcare Research, Providence, Rhode Island, United States; Brown University, Providence, Rhode Island, United States; University of Alabama at Birmingham, Birmingham, Alabama, United States

## Abstract

There is limited information regarding Alzheimer’s Disease and Related Dementia Diagnosis (ADRD) in Puerto Rico (PR), making it difficult to draw comparisons of the quality of care for people with ADRD between PR and the US mainland. In fact, data from the Mapping Medicare Disparities by Population Tool from Medicare only shows prevalence rates for fee-for-service Medicare enrollees. Given that about 90% of PR beneficiaries are enrolled in Medicare Advantage (MA), these rates may not reflect the full extent of older adults with ADRD on the island. Thus, the purpose of the study was to compare ADRD prevalence rates among Hispanic MA enrollees in PR to those of their US mainland counterparts. We used 20% Medicare beneficiaries (carrier file claims) and applied up to one-year lookback period. We focused on adults who were 65 and older. Our study sample included 123,609 and 547,978 Hispanic MA enrollees in PR and the US mainland in 2019. Approximately, 54% and 56% of enrollees in PR and the US mainland were female. The ADRD rates were 15% compared to 9% for PR and enrollees in the mainland. Our results show that MA enrollees in PR have six percentage points higher ADRD rates than their US counterparts. This highlights the importance of reporting ADRD rates, including MA beneficiaries. Further research on the characteristics and healthcare utilization for the people with ADRD in PR is required. This information is needed to improve the care and supports for people with ADRD and their caregivers (ASPE, 2022).

